# Assessment of Patient Empowerment - A Systematic Review of Measures

**DOI:** 10.1371/journal.pone.0126553

**Published:** 2015-05-13

**Authors:** Paul J. Barr, Isabelle Scholl, Paulina Bravo, Marjan J. Faber, Glyn Elwyn, Marion McAllister

**Affiliations:** 1 Dartmouth College, Lebanon, United States of America; 2 University Medical Center Hamburg-Eppendorf, Hamburg, Germany; 3 Cardiff University, Cardiff, United Kingdom; 4 Pontificia Universidad Católica de Chile, Santiago, Chile; 5 Radboud University Medical Center, Scientific Institute for Quality of Healthcare, Nijmegen, The Netherlands; Canadian Agency for Drugs and Technologies in Health, CANADA

## Abstract

**Background:**

Patient empowerment has gained considerable importance but uncertainty remains about the best way to define and measure it. The validity of empirical findings depends on the quality of measures used. This systematic review aims to provide an overview of studies assessing psychometric properties of questionnaires purporting to capture patient empowerment, evaluate the methodological quality of these studies and assess the psychometric properties of measures identified.

**Methods:**

Electronic searches in five databases were combined with reference tracking of included articles. Peer-reviewed articles reporting psychometric testing of empowerment measures for adult patients in French, German, English, Portuguese and Spanish were included. Study characteristics, constructs operationalised and psychometric properties were extracted. The quality of study design, methods and reporting was assessed using the COSMIN checklist. The quality of psychometric properties was assessed using Terwee’s 2007 criteria.

**Findings:**

30 studies on 19 measures were included. Six measures are generic, while 13 were developed for a specific condition (N=4) or specialty (N=9). Most studies tested measures in English (N=17) or Swedish (N=6). Sample sizes of included studies varied from N=35 to N=8261. A range of patient empowerment constructs was operationalised in included measures. These were classified into four domains: patient states, experiences and capacities; patient actions and behaviours; patient self-determination within the healthcare relationship and patient skills development. Quality assessment revealed several flaws in methodological study quality with COSMIN scores mainly fair or poor. The overall quality of psychometric properties of included measures was intermediate to positive. Certain psychometric properties were not tested for most measures.

**Discussion:**

Findings provide a basis from which to develop consensus on a core set of patient empowerment constructs and for further work to develop a (set of) appropriately validated measure(s) to capture this. The methodological quality of psychometric studies could be improved by adhering to published quality criteria.

## Introduction

Patient empowerment is gaining greater international importance in healthcare [1]. Reflecting the shift in Western culture towards increasing consumerism and individualism, institutional culture in healthcare is slowly moving away from an ethic of paternalism towards an ethic of empowering patients to make informed decisions. This is demonstrated by interest in developing and implementing more equitable and collaborative approaches to the healthcare relationship, including shared decision-making [2–5]. There is some limited evidence that patient empowerment can improve cost-effective use of health services [1]. Self-care interventions for long-term conditions, sometimes called patient empowerment programmes, have been shown to improve mental health, doctor-patient communication, healthy eating, and patient self-efficacy [6,7], although the capacity of existing outcome measures to capture the patient benefits of these programmes has been questioned [8].

In the UK, government health policy declared in “High Quality Care for All” [9] committed the National Health Service (NHS) to patient empowerment. Since then, initiatives designed to provide NHS patients with greater choice and control over their own healthcare have been developed and implemented, for example Personal Health Budgets and Personalised Care Plans. Furthermore, following the 2011 Health & Social Care Bill in England, there are moves to link NHS funding to performance against a range of existing and new quality measures that include patient reported outcome measures (PROMs). Similarly, in the USA, the quality of patient centered care, including patient empowerment, may soon be linked to remuneration and improved legal protection for healthcare professionals [10,11].

However, despite this, there is no agreement about the best way to demonstrate that patients have, indeed, been empowered. The concept of patient empowerment has not been consistently operationalised because there is no consensus about how the term should be defined [12]. There is no universally accepted measure of patient empowerment that can be used to evaluate and compare patient empowerment initiatives across different healthcare services, although some quite generic patient empowerment measures have been published recently [13,14]. A number of condition-specific and specialty-specific patient empowerment measures have also been published, for example, the Empowerment Scale (mental health) [15], the Diabetes Empowerment Scale [16], the Patient Empowerment Scale (cancer) [17] and the Genetic Counselling Outcome Scale (clinical genetics) [18]. A 2009 systematic review of questionnaires measuring health-related empowerment identified 50 questionnaires purporting to measure health-related empowerment, and rated these in terms of reliability and validity [19]. This review did not assess the methodological quality of the included studies, provided only a limited assessment of the psychometric properties of included measures [20–25], and included measures that were intended for completion by non-patients, e.g. parents or family members.

A brief review of self-report questionnaires capturing patient empowerment to date suggests that available measures in this area have been developed independently, with scale content informed by different theoretical frameworks [12]. The constructs captured by measures in this brief review were not the same, although there are some areas of identifiable theoretical overlap across some of these measures that relate to decision-making, control and self-efficacy [12]. We do not know at present whether this is because different constructs are important for different conditions, or because they were developed independently without a generic theoretical framework of patient empowerment for guidance. This apparent heterogeneity across measures of patient empowerment means that approaches, interventions and policies designed to empower patients in healthcare cannot be evaluated on the basis of how effective they are at achieving this goal because the goal itself is not clear, and there is little agreement about how to measure it.

Given both the interest in patient empowerment and the need for high quality patient reported measurement, we set out to conduct a systematic review with a focus on patient-reported measures of patient empowerment that could be used as PROMs. This review is the first to apply current published methodological standards for conducting systematic reviews of measurement instruments [22,24,26] and guidelines to assess the psychometric properties of the identified measures of patient empowerment [23]. The key aims of our review were as follows: to identify measures of patient empowerment that have been developed and psychometrically tested; to assess the quality of existing patient empowerment measures; and finally to describe the conceptual domains captured by existing measures of patient empowerment.

## Methods

### 1. Protocol and registration

The protocol for this systematic review was registered on PROSPERO: http://www.crd.york.ac.uk/PROSPERO/display_record.asp?ID=CRD42013003961#.U2zSMWcU-L0

### 2. Eligibility criteria

Peer-reviewed studies that reported the psychometric properties of a patient reported measure or PROM that assessed patient empowerment were retrieved. The aim was to include measures of patient empowerment and measures of related constructs, including enablement, activation, perceived control, capability and independence. To be included, the measure had to have been tested in a sample of adult patients in a healthcare setting and one aim of the study had to be to assess psychometric properties of the measure. Articles in English, French, German, Portuguese and Spanish were all retrieved. Studies that lacked a psychometric assessment, were not tested in a sample of adult patients or were designed for completion by children or other relatives or carers were excluded.

### 3. Information sources

To achieve a highly sensitive search strategy, databases were searched from their inception to Sept 15, 2012 using a number of Medical Subject Headings (MeSH) and keywords in four domains: (i) patient (ii) empowerment, (iii) measures and (iv) psychometrics (S1 Fig). Databases searches were conducted in MEDLINE, EMBASE, Cochrane, Web of Science and ASSIA. A secondary search of the reference sections of included papers and identified review articles was also conducted.

### 4. Search, study selection, & data collection

The electronic search strategies used are available in S1 Fig Titles and abstracts retrieved from the database searches were equally distributed to four team members (PB, IS, PBr, MMc) for independent screening. To ensure screening quality and consistency, the first 15% of each reviewer’s titles and abstracts were rescreened by another member of the team with a comparison of included and excluded titles made; disagreements were resolved by discussion. This ensured that exclusion criteria were applied consistently for the remaining 85% of titles and abstracts. The standardised data extraction procedure was first piloted and then implemented by four members of the study team (PB, IS, PBr, MMc). Data extracted included the study aims, patient population, setting, purpose and description, including psychometric properties of the patient reported measure under evaluation.

### 5. Study quality and risk of bias appraisal

The Center for Reviews and Dissemination (CRD) and the Preferred Reporting Items for Systematic Reviews and Meta-Analyses (PRISMA) recommends the use of checklists to appraise study quality [6]. We undertook two assessments of quality with two distinct aims: Firstly, to evaluate the methodological quality of the included studies. This was achieved using the COnsensus-based Standards for the selection of health Measurement Instruments (COSMIN) criteria. However, the COSMIN criteria do not provide an assessment of the psychometric properties of the instruments themselves. To address this, we used criteria developed by Terwee et al. (2007) [23].

The COSMIN criteria were used to assess methodological quality of the included studies. The COSMIN checklist evaluates nine psychometric properties (‘A’ through ‘I’: A = internal consistency, B = reliability; C = measurement error, D = content validity, E = structural validity, F = hypothesis testing; G = cross-cultural validity, H = criterion validity, I = responsiveness). Each box comprises items evaluating methodological quality, such as appropriateness of sample size and psychometric statistic(s) generated. Each COSMIN item can be rated as ‘Excellent’, ‘Good’, ‘Fair’ or ‘Poor’, with a description provided for how to attribute a rating to each COSMIN item. An additional box was used to assess requirements for studies that used Item Response Theory (IRT). Interpretability and generalisability were also assessed for each of the ten boxes that investigated methodological quality.

To apply the COSMIN criteria four steps were followed: 1) Identify what properties (boxes) were assessed in the paper and select these boxes, 2) determine if classical test theory (CTT) or IRT was used, 3) evaluate methodological quality of studies identified in step 1 and 4) assess the generalisability of results from the studies on properties identified in step 1. Further details of how the COSMIN criteria are applied are available online at http://www.cosmin.nl/. The ‘worst score count’ is the method of assessment for COSMIN boxes A to I, i.e. the lowest rating on an item from any one box was considered the overall score for that box. Therefore where the majority of items within the box are considered ‘good’, and one item is marked as ‘fair’ the overall quality of that box is considered ‘fair’. For COSMIN criteria, the overall proportion of included studies that achieved a rating of ‘Excellent’, ‘Good’, ‘Fair’ or ‘Poor’, is presented for each methodological element (box) examined. For interpretability and generalisability boxes there is no possibility to aggregate the extracted data into a sum score, therefore these sections will be summarised qualitatively.

Criteria developed by Terwee et al [23] to appraise the psychometric quality of questionnaires were used to assess the quality of the measures identified in this study. Criteria assessed were: internal consistency, content validity, criterion validity, construct validity, reproducibility, responsiveness, floor and ceiling effects and interpretability. Items were rated as positive ‘+’, intermediate ‘?’, negative ‘-‘, or no information ‘ _ ‘ (Further detail in Terwee et al [23]). The authors [23] suggest presenting the results in the form of a table rather using an ‘overall quality score’. Such a score would assume equal importance for each psychometric property, which in practice is not the case. In addition, as recommended by Terwee et al [23] criterion validity was not assessed in this study using either COSMIN or Terwee et al criteria because there is no gold standard comparison for measures of patient empowerment.

At the full paper screen, the included articles were again distributed equally to four team members (PB, IS, PBr and MMc) for assessment of the methodological quality of studies and the quality of included measures. To ensure consistency in application of the COSMIN criteria, a fifth team member (MF) independently applied the COSMIN criteria to a random sample of 25% of included papers at the start of data extraction. Any disagreements were resolved through team discussion prior to extracting data for the remaining 75% of articles. One of the included studies was conducted by the senior author (MMc). To avoid any intellectual conflict of interest, this study was neither extracted nor rated by MMc.

### 6. Data analysis and synthesis of results

The key characteristics of the studies and the quality of the studies were combined in a narrative summary and tabulated according to CRD guidelines [27]. In addition, the key constructs (subscales, or definitions where subscales were not reported) measured by each PROM were recorded and these were analysed to identify general themes that emerged regarding constructs captured by the included measures of patient empowerment.

## Results

### 1. Included studies

**Fig 1:**
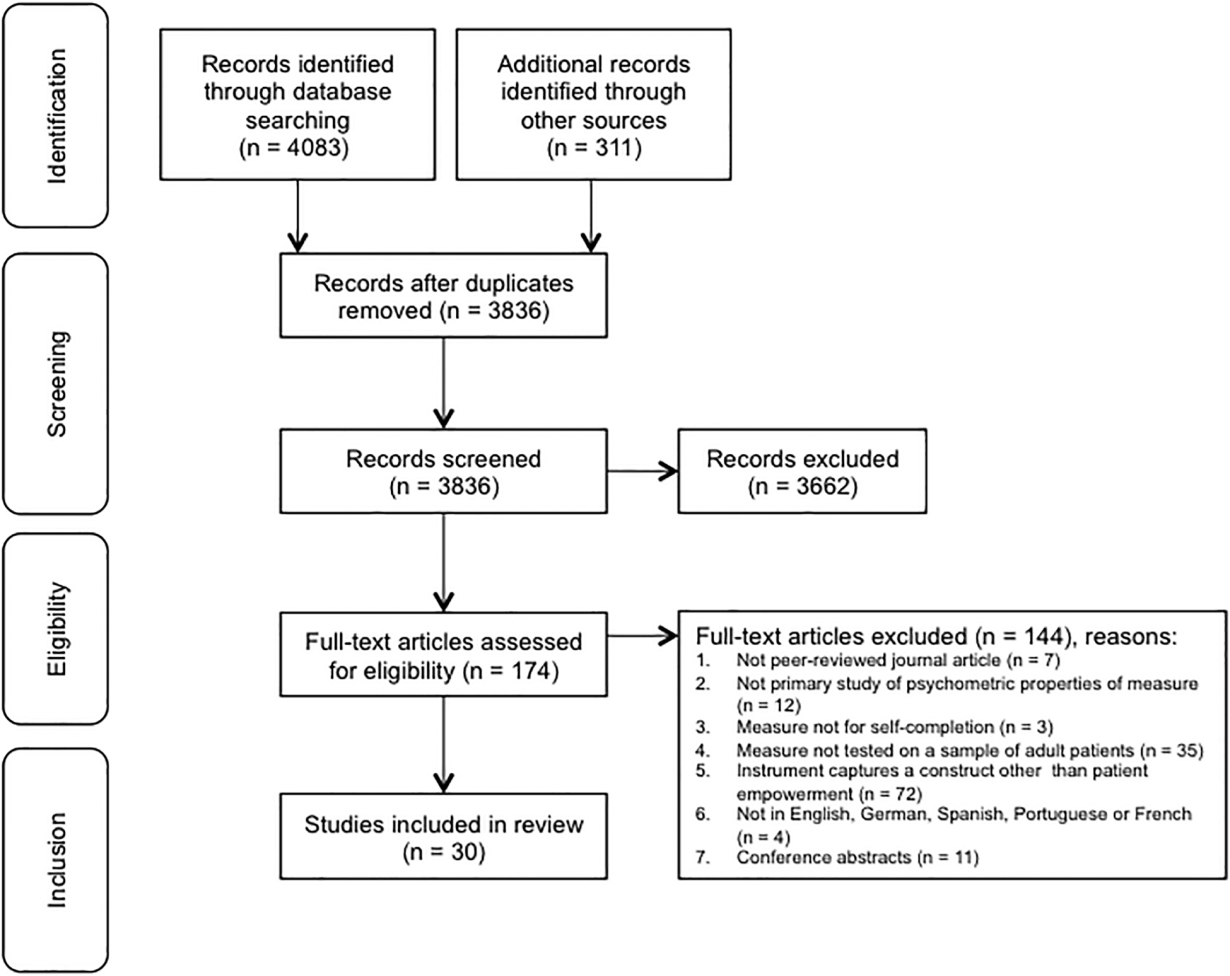
PRISMA flow chart of study selection.

Electronic searches identified 4083 records and the secondary search generated 311 additional records. After removing duplicates, 3836 records remained. Title and abstract screening resulted in exclusion of 3662 records. The remaining 174 full-text articles were retrieved and assessed for eligibility. The original aim had been to identify measures of patient empowerment, enablement, activation, perceived control, capability and independence. However, following identification of full-text articles, a decision was made to include only measures that specifically stated that they were designed to measure patient empowerment. Focusing only on measures that purport to capture patient empowerment enabled more clarity to be provided regarding the constructs used to operationalise patient empowerment and removed the ambiguity of including related, but subtly different constructs. This led to the inclusion of 30 studies. The main reasons for exclusion were that the measure did not purport to capture a construct called patient empowerment (N = 72) or was not tested in a sample of adult patients (N = 35). The 72 measures excluded because they captured other constructs included 12 measures capturing patient enablement, 16 measures capturing patient activation, 27 measures capturing perceived control and 17 measures capturing other constructs. No articles were identified by the search strategy that captured either capability or independence. Fig 1 provides the study PRISMA flow chart with the complete list of reasons for exclusion at the stage of eligibility assessment.

### 2. Overview of studies

Table 1 gives an overview of included measures and Table 2 provides an overview of included studies. In total 30 studies were included in the review, reporting on 19 different measures. Fourteen measures were developed specifically to assess patient empowerment and in five measures patient empowerment was included as one subscale of a larger questionnaire. Six measures are generic, while the other 13 were developed for a specific condition (N = 4) or to be used within a specific specialty (N = 9). Half of the studies (N = 15) were reporting on initial development and validation of the measure, and the rest were reporting on further development (including translation) of an existing measure. Some studies reporting initial development of a measure were excluded (e.g. Empowerment Scale (mental health)) because the initial development and validation study did not test the measure with patients [15]. However, studies further developing the measure with patient samples are included. Ten of the studies were from the US, six from Sweden, four from the UK, three from China, three from Australia, and one each from Japan, Italy, Iran and Iceland. Most studies reported on the validation of measures in English (N = 17) or Swedish (N = 6). The number of items in included measures ranged from 5 to 42 items. Of the 19 measures, ten use a five-point Likert scale for response categories. Sample sizes of the studies vary from N = 35 to N = 8261 patients. Conditions affecting the patients included in the test samples varied, with several studies conducted with samples of patients affected by a mental health condition (N = 10) [28–37] or diabetes (N = 7) [16,38–43].

**Table 1:** Overview of included measures.

Measure (Author)	Target population	Items	Sub-scales	Response scale	Language*
Patient Empowerment Scale #1—Original (Faulkner, 2001) [13]	All patients (generic)	40	No subscales reported; designed to capture feelings of control in hospital environments catering for older people by asking about empowering and disempowering behaviours of hospital staff	3-Point Likert Scale	English
Kim Alliance Scale—Original (Kim et al, 2001) [46]	All patients (generic)	30	Four subscales: Collaboration, Communication, Integration, Empowerment	4-Point Likert Scale	English
Kim Alliance Scale—Revised (Kim et al, 2008) [47]	All patients (generic)	16	Four subscales: Collaboration, Communication, Integration, Empowerment	4-Point Likert Scale	English
Treatment Related Empowerment Scale (TES)—Original (Webb et al, 2001) [45]	All patients (generic)	10	Four subscales: Choice, Decision making, Communication, Satisfaction	5-Point Scale	English
Health Education Impact Questionnaire (HEIQ)—Original (Osborne et al, 2007) [44]	All patients (generic)	42	Eight subscales: Positive active engagement in life, Health directed behavior, Skill & technique acquisition, Constructive attitudes & approaches, Self-monitoring & insight, Health services navigation, Social integration & support, Emotional wellbeing	Not specified	English
Scale developed by Bann et al.—Original (Bann et al, 2010) [49]	All patients (generic)	5	No subscales reported (Aim was to create three scales: Perceived provider support, Patient-centered care (PCC) and Empowerment)	3-Point Scale	English
Health Care Empowerment Inventory (HCEI)—Original (Johnson et al, 2012) [52]	All patients (generic)	8	Two subscales: HCE ICCE: informed, committed, collaborative & engaged, HCE TU: tolerance for uncertainty	5-Point Likert Scale	English
Empowerment Scale—Original (Wowra & McCarter, 1999) [29]	Mental health patients	28	Five subscales: Self-esteem & self-efficacy, Optimism & control over the future, Power & powerlessness, Activism & autonomy, Righteous anger	4-point Likert Scale	English Japanese Swedish
Empowerment Scale—Version 2 (Corrigan et al. 1999) [36]	Mental health patients	25	Two super-ordinate factors: (i) *Self-orientation*: three sub-scales: Self-esteem, Self-efficacy, Optimism (ii) *Community orientation*: three sub-scales: Community action, Powerlessness, Effecting change	4-point Likert Scale	English
Health Promotion Intervention Questionnaire—Original (Svedberg et al, 2007) [33]	Mental health patients	19	Four subscales: Alliance, Empowerment, Educational support, Practical support	5-Point Likert Scale	Swedish
Empowerment Questionnaire for Inpatients (EQuIP)—Original (Lopez et al, 2010) [37]	Mental health patients	16	No subscales reported (Expected sub-scales were information, choice, and communication, but CFA did not confirm this and EFA was not conducted)	4-Point Likert Scale	English
Consumer Evaluation Of Mental Health Services (CEO-MHS)—Original (Oades et al, 2011) [34]	Mental health patients	26	Two subscales: Empowerment (consumers’ sense of control over their illness, treatment and stigma), Dehumanization	5-Point Likert Scale	English
Inpatient Consumer Survey—Original (Ortiz & Schacht, 2012) [35]	Mental health patients	28	Six subscales: Outcome, Dignity, Rights, Participation, Environment, Empowerment (patient choice & communication)	5-Point Likert Scale	English
Diabetes Empowerment Scale—Original (Anderson et al, 2000) [16]	Diabetes patients	28	Three subscales: Managing the psychosocial aspects of diabetes, Assessing dissatisfaction & readiness to change, Setting & achieving diabetes goals	5-Point Likert Scale	English Icelandic Persian
Diabetes Empowerment Scale—Version 2 (Shiu et al, 2003) [39]	Diabetes patients	20	Five subscales: Overcoming barriers, Determining suitable methods, Achieving goals, Obtaining support, Coping	5-Point Likert Scale	Chinese
Diabetes Empowerment Scale—Version 3 (Leksell et al, 2007) [38]	Diabetes patients	23	Four subscales: Goal achievement, Self awareness, Stress management, Readiness to change	5-Point Likert Scale	Swedish
Chinese Diabetes Empowerment Process Scale (C-DEPS)—Original (Chen et al, 2011) [43]	Diabetes patients	15	Four subscales: Mutual participation, Raising awareness, Providing necessary information, Open communication	5-Point Likert Scale	Chinese
Patient Empowerment Scale #2—Original (Bulsara et al, 2006) [17]	Cancer patients	28	No subscales reported; designed to capture ability to accept illness, develop coping strategies, regain a sense of control	4-Point Likert Scale	English
Cyber Info-Decisional Empowerment Scale (CIDES)—Original (Seckin, 2011) [53]	Cancer patients	7	No Subscales reported (Aim was to capture extent to which cyber-information provide knowledge about cancer, treatment & whether to obtain second opinion; informs about research and health services; facilitates decision-making regarding treatment)	5-Point Likert Scale	English
Genetic Counseling Outcome Scale—Original (McAllister et al, 2011) [18]	Clinical genetics patients	24	No Subscales reported (Aim was to create five subscales: cognitive control, decisional control, behavioural control, emotional regulation, hope for the future; EFA performed but unstable)	7-Point Likert Scale	English
Parents’ Postnatal Sense of Security (PPSS)—Mother version—Original (Persson et al, 2007) [48]	Postnatal care patients	18	Four subscales: Sense of midwifes'/nurses' empowering behavior, Sense of general wellbeing, Sense of affinity with the family, Sense that breast feeding was manageable	4-Point Likert Scale	Swedish
Parents’ Postnatal Sense of Security (PPSS)—Father Version—Original (Persson et al, 2007) [48]	Postnatal care patients	13	Four subscales: Sense of midwifes'/nurses' empowering behavior, Sense of the mother's general wellbeing including breast feeding, Sense of affinity with the family, Sense of general wellbeing	4-Point Likert Scale	Swedish
Psoriasis Empowerment Enquiry in the Routine practice questionnaire (PEER)—Original Pagliarello et al (2010) [50]	Psoriasis patients	12	Three subscales: Knowledge, Experience, Skills	5-Point Likert Scale	Italian
The Swedish Rheumatic Disease Empowerment Scale (SWE-RES-23)—Original (Arvidsson et al, 2012) [51]	Rheumatic disease patients	23	5 Subscales: Goal achievement & overcoming barriers to goal achievement, Self-knowledge, Managing stress, Assessing dissatisfaction & readiness to change, Support for caring	5-Point Likert Scale	Swedish

* Language of measures used by included studies.

**Table 2:** Characteristics of included studies.

Author (year), Scale (target population)	Country	Study population	Sample characteristics
Faulkner (2001) [13], Patient Empowerment Scale # 1 (All patients, generic)	UK	Older hospitalized people	Pre-test sample: n = 78; main sample: n = 102
Kim et al. (2001) [46], Kim Alliance Scale (KAS) (All patients, generic)	USA	Nurses as patients	n = 68
Kim et al. (2008) [47], Kim Alliance Scale Revised (KAS-R) (All patients, generic)	USA	Patients of two outpatient clinics serving military family members and retirees	Sample 1: n = 304, 21% male, mean age 39.2 years, SD 12.8; Sample 2: n = 297, 20% male, mean age 40.7 years, SD 12.6
Webb et al. (2001) [45], Treatment Related-Empowerment Scale (TES) (All patients, generic)	UK	Patients with advanced HIV	n = 43, 93% male, mean age 39.6 years, SD 7.7
Osborne et al. (2007) [44], Health Education Impact Questionnaire (HEIQ) (All patients, generic)	Australia	Both samples had a wide range of chronic diseases	Sample 1: members of the Arthritis foundation, n = 591, 84% female, mean age 62, SD 13; Sample 2: broader population, n = 598, 58% female, mean age 61, SD 14
Bann et al. (2010) [49], Scale developed by Bann et al. (2010) (All patients, generic)	USA	Patients with various health problems receiving complementary and alternative medicine (CAM)	Pre-test sample: n = 44; main sample: n = 216, 85% female, age: 29% < 44, 28% 45–54, 43% > 54
Johnson et al. (2012) [52], Health Care Empowerment Inventory (HCEI) (All patients, generic)	USA	HIV infected patients	Sample 1: n = 275, 100% male, mean age 46.9, SD 9.6; Sample 2: n = 370, 78.2% male, mean age, 45.2, SD 8.2
Wowra & McCarter (1999) [29], Empowerment Scale version 1 (Mental health patients)	USA	Outpatients of mental health services	n = 283, 67% female, age 62% range 36 55
Corrigan et al. (1999) [36], Empowerment Scale version 2 (Mental health patients)	USA	Consumers of a university partial hospitalization program, all with a diagnosis of severe mental illness	n = 35, 35.1% female, mean age 33.1 years, SD 9.2
Hansson & Bjorkman (2005) [28], Empowerment Scale version 1 (Mental health patients)	Sweden	Patients with severe mental illnesses	n = 92, 53% female, mean age 47 years, range 29–68
Yamada & Kuzuki (2007) [31], Empowerment Scale version 1 (Mental health patients)	Japan	Patients with chronic schizophrenia	n = 72, 40% female, mean age 41.7 years, SD 10.7
Rogers et al. (2010) [30], Empowerment Scale version 1 (Mental health patients)	USA	Consumers of mental health services with a diagnosis of severe mental disorder	n = 1827, 64% female, mean age 41 years, range 18–72
Svedberg et al. (2007)* [33], Health Promotion Intervention Questionnaire (Mental health patients)	Sweden	Patients in contact with mental health services	n = 135, 64% female, mean age 41 years, range 18–72
Svedberg et al. (2008)* [32], Health Promotion Intervention Questionnaire (Mental health patients)	Sweden	Patients in contact with mental health services	Pre-test sample: n = 31; main sample: n = 135, 64.4% female, mean age 41 years, range 18–72
Lopez et al. (2010) [37], Empowerment Questionnaire for Inpatients (EQuIP) (Mental health patients)	UK	Patients older than 65 years with a functional psychiatric diagnosis	n = 87, 61% female, mean age 73.7 years, SD 5.9, range 65–87
Oades et al. (2011) [34], Consumer Evaluation Of Mental Health Services (CEO-MHS) (Mental health patients)	Australia	Mental health services consumers	n = 202, 49% female, mean age 40.9 years, SD 13.06, range 16–83
Ortiz & Schacht (2012) [35], Inpatient Consumer Survey (Mental health patients)	USA	Individuals receiving psychiatric care	Sample 1: n = 8229, 65% male, 78% aged between 18–54 years; Sample 2: n = 8261, 65% male, 78% aged between 18–54 years
Anderson et al. (2000) [16], Diabetes Empowerment Scale version 1 (Diabetes patients)	USA	Type 1 or 2 diabetes patients	n = 375, 45% male, mean age 50.4 years, SD 15.8
Shiu et al. (2003) [39], Diabetes Empowerment Scale version 2 (Diabetes patients)	China	Type 1 or 2 diabetes patients	Pre-test sample: n = 31; main sample: n = 207, 52.2% female, mean age 53 years, SD 12.4
Shiu et al. (2006)[40], Diabetes Empowerment Scale version 2 (Diabetes patients)	China	Type 1 or 2 diabetes patients	n = 189, 49% male, mean age 52.26 years, SD 12.43
Leksell et al. (2007)[38], Diabetes Empowerment Scale version 3 (Diabetes patients)	Sweden	Type 1 or 2 diabetes patients	n = 195, 51.6% male, mean age 59.4 years, range 22–90
Sigurdardottir & Jonsdottir (2008)[41], Diabetes Empowerment Scale version 1, (Diabetes patients)	Iceland	Diabetes patients	n = 90, 53.3% female, mean age 38.11 years, SD 11.11
Tol et al. (2012)[42], Diabetes Empowerment Scale version 1 (Diabetes patients)	Iran	Type 2 diabetes patients	Pre-test sample: n = 14; main sample: n = 160, 72.9% female, mean age 50.23 years, SD 11.0, range 19–83
Chen et al. (2011) [43], Chinese Diabetes Empowerment Process Scale (C-DEPS) (Diabetes patients)	China	Type 1 or 2 diabetes patients	Pre-test sample: n = 20; main sample: n = 211, 50.2% female, mean age 59.3 years, SD 13.4, range 20–87
Bulsara et al. (2006) [17], Patient Empowerment Scale (PES) # 2 (Cancer patients)	Australia	Cancer patients	n = 113
Seckin (2011) [53], Cyber Info-Decisional Empowerment Scale (CIDES) (Cancer patients)	USA	Cancer patients	n = 350, 72.9% female, mean age 50.23 years, SD 11.0, range 19–83
McAllister et al. (2011) [18], Genetic Counseling Outcome Scale (Clinical genetics patients)	UK	Patients with genetic conditions	Sample 1: n = 527, 79.5% female, 35.2% aged 17–40 years; 33.4% aged 40–49 years; 31.4% aged 50–80 years; Sample 2: n = 395, 74.4% female, age range 18–79
Persson et al. (2007) [48], Parents’ Postnatal Sense of Security (PPSS) (Postnatal care patients)	Sweden	Parents 1 week post child birth	Sample 1: n = 113 mothers, mean age 29.8 years; Sample 2: n = 99 fathers, mean age 32.4 years
Pagliarello et al. (2010) [50], Psoriasis Empowerment Enquiry in the Routine practice questionnaire (PEER) (Psoriasis patients)	Italy	Psoriasis patients	n = 223, 50.7% female, mean age 45.5 years, SD 15.5
Arvidsson et al. (2012) [51], The Swedish Rheumatic Disease Empowerment Scale (SWE-RES-23) (Rheumatology patients)	Sweden	Patients with rheumatic diseases	Pre-test sample: N = 58; main sample: n = 260, 75% female, mean age 54 years

SD = standard deviation

* The two papers on the Health Promotion Intervention Questionnaire report on different research questions (assessment of different psychometric properties) of the same study (same sample size)

^#^ We found two measures named Patient Empowerment Scale that are not related to each other

### 3. Constructs captured by included measures

Details of the subscales used to capture patient empowerment were extracted from the included articles (See Table 1). Six articles did not report any subscales; for these, we have included some information on what the measure was intended to capture. Constructs captured by the included articles illustrate a diversity of conceptualisations of patient empowerment, captured by a wide range of different subscales (See Tables 1 & 3).

**Table 3:** Domains captured across generic and condition- or specialty-specific measures.

Measure (target population)	Domain 1 Patient states, experiences and capacities	Domain 2 Patient actions and behaviours	Domain 3 Patient self-determination within the healthcare relationship	Domain 4 Developing patient skills
*Patient Empowerment Scale # 1 [13] (All patients, generic)	**+**	**-**	**-**	**-**
Kim Alliance Scale [46,47] (All patients, generic)	**+**	**+**	**+**	**-**
Treatment Related-Empowerment Scale (TES) [45] (All patients, generic)	**+**	**-**	**+**	**-**
Health Education Impact Questionnaire [44] (All patients, generic)	**+**	**+**	**-**	**+**
*Scale developed by Bann et al. (2010) [49] (All patients, generic)	**+**	**+**	**+**	**+**
Health Care Empowerment Inventory (HCEI) [52] (All patients, generic)	**+**	**-**	**+**	**-**
Empowerment Scales (Original) [28–31] (Mental health patients)	**+**	**+**	**-**	**-**
Empowerment Scales (Version 2) [36] (Mental health patients)	**+**	**+**	**-**	**-**
Health Promotion Intervention Questionnaire [32,33] (Mental health patients)	**-**	**-**	**+**	**-**
*Empowerment Questionnaire for Inpatients (EQuIP) [37] (Mental health patients)	**+**	**-**	**+**	**-**
Consumer Evaluation Of Mental Health Services (CEO-MHS) Scale [34] (Mental health patients)	**+**	**-**	**-**	**-**
Inpatient Consumer Survey (Mental health patients)	**-**	**-**	**+**	**-**
Diabetes Empowerment Scale (Original) [16,41,42] (Diabetes patients)	**+**	**+**	**-**	**-**
Diabetes Empowerment Scale (Version 2) [39,40] (Diabetes patients)	**+**	**+**	**-**	**-**
Diabetes Empowerment Scale (Version 3) [38] (Diabetes patients)	**+**	**+**	**-**	**-**
Chinese Diabetes Empowerment Process Scale (C DEPS) [43] (Diabetes patients)	**+**	**-**	+	**-**
*Patient Empowerment Scale #2 [17] (Cancer patients)	+	-	**-**	**-**
*Cyber Info-Decisional Empowerment Scale (CIDES) [53] (Cancer patients)	+	**-**	+	**-**
*Genetic Counseling Outcome Scale [18] (Clinical Genetics patients)	+	**-**	**-**	**-**
Parents’ Postnatal Sense of Security (PPSS) [48] (Postnatal patients)	+	**-**	**+**	-
Psoriasis Empowerment Enquiry in the Routine practice questionnaire (PEER) [50] (Psoriasis patients)	+	-	**-**	+
The Swedish Rheumatic Disease Empowerment Scale (SWE-RES-23) [51] (Rheumatology patients)	+	-	**-**	**-**

* Six measures did not report sub-scales. For these measures, the domain(s) captured were inferred by examining the definition of patient empowerment that the authors claimed to capture.

**Domain 1:** Self-efficacy; Self-esteem; Self-confidence; Satisfaction; Stigma; Commitment & engagement; Self-monitoring, self-knowledge & insight/ awareness; Optimism / hope; Perceived (sense of) control; Righteous anger; Acceptance; Enablement; Readiness to change; Power / powerlessness; Tolerance for uncertainty; Sense of affinity with the family; Motivation to advocate for oneself; Having knowledge / Information / being informed; Emotional regulation / wellbeing; constructive attitudes and approaches; Autonomy.

**Domain 2**: Learn from past experience; Positive active engagement with life; Health directed behaviour / manage and improve own health; Health service navigation; Social integration; Effecting change; Community activism; Coping (strategies including obtaining support); Managing psychosocial aspects of disease; Setting & achieving disease-related goals (including determining suitable methods and overcoming barriers); Stress management.

**Domain 3:** Power-sharing/collaboration/mutual participation; Mutual / Patient decision-making; Patient choice / self-determination self; Communication.

**Domain 4:** Developing skills / skills acquisition; Sense of healthcare providers empowering behaviour.

There was no clear consensus across the included articles about what patient empowerment comprises. Each included measure captured a different conceptualisation of patient empowerment, with approximately 38 distinct constructs identifiable. A synthesis of constructs captured by the included measures is shown in Table 3, grouped into four domains. Constructs ranged from patient empowerment conceptualised as:

Domain 1: Patient states, experiences and capacities (21 constructs)
Domain 2: Patient actions and behaviours (11 constructs)
Domain 3: Patient self-determination within the healthcare relationship (four constructs)
Domain 4: Patient skills development (two constructs).

Fifteen constructs were captured by one measure only. Another 14 constructs were captured by at least one generic measure and at least one specialty or condition-specific measure.

### 4. Quality of design, methods and reporting

Table 4 provides an overview of the assessment of the methodological quality of included studies using the COSMIN criteria [22,24,26]. Detailed results for the COSMIN checklist with 4-point scale ratings are shown in S1 Table. While most studies used classical test theory (CTT), one study used item response theory (IRT) [17] and one used both CTT and IRT [44]. Only one study earned a rating of excellent on an aspect of methodological quality [34]. Included studies assessed a median of four out of the nine COSMIN criteria. Across the included measures only an average of three psychometric properties, of the nine possible psychometric properties, were assessed, of which only one, on average, received a positive score. All but three studies [31,33,36] reported internal consistency and could be rated using COSMIN box A. Ratings for internal consistency were either poor [13,17,18,28,30,34,35,37,41,44,45] or fair [16,29,32,38–40,42,43,46–53]. Reliability (box B) was only assessed in six studies, either resulting in poor [32,37,39] or fair ratings [18,36,43]. Measurement error was not reported in any study and could therefore not be rated with COSMIN box C. Content validity (box D) was assessed by most studies with a high variability of scores, ranging from several studies rated as poor [13,16,28,35,37,38,45–47,49–52] to one single study rated as excellent [34]. Structural validity (box E) was also assessed by most studies with only the study on the Genetic Counseling Outcome Scale [18] being rated as good and all other studies receiving poor [28,30,31,34,36,37,41] or fair ratings [16,17,29,32,35,38–40,42–44,46–53]. Regarding hypothesis testing (box F) studies were either rated as poor [16,18,28,30,31,35–38,43,45–47] or fair [29,32,33,39–42,49,51–53]. Testing of cross-cultural validity (box G) was only applicable for five studies, but was rated poor for three of those studies [28,38,41] and fair for the other two studies[39,42]. Responsiveness (box I) was only assessed in one study [18] and was rated as fair.

**Table 4:** Quality of design, methods and reporting of studies on psychometric properties (COSMIN ratings).

Measure (target population)	Authors (Year)	IRT or CTT	Score IRT	A	B	C	D	E	F	G	I
Patient Empowerment Scale (All patients, generic)	Faulkner (2001) [13]	CTT		0			0				
Kim Alliance Scale (All patients, generic)	Kim et al. (2001) [46]	CTT		+			0	+	0		
Kim Alliance Scale Revised (All patients, generic)	Kim et al. (2008) [47]	CTT		+			0	+	0		
Treatment-Related Empowerment Scale (All patients, generic)	Webb et al. (2001) [45]	CTT		0			0		0		
Health Education Impact Questionnaire (All patients, generic)	Osborne et al. (2007) [44]	CTT & IRT	++	0			+	+			
No name (Scale developed by Bann et al) (All patients, generic)	Bann et al. (2010) [49]	CTT		+			0	+	+		
Health Care Empowerment Inventory (All patients, generic)	Johnson et al. (2012) [52]	CTT		+			0	+	+		
Empowerment Scale version 1 (Mental health patients)	Wowra & McCarter (1999) [29]	CTT		+				+	+		
Empowerment Scale version 1 (Mental health patients)	Hansson & Bjorkman (2005) [28]	CTT		0			0	0	0	0	
Empowerment Scale version 1 (Mental health patients)	Yamada & Kuzuki (2007) [31]	CTT						0	0		
Empowerment Scale version 1 (Mental health patients)	Rogers et al. (2010) [30]	CTT		0				0	0		
Empowerment Scale version 2 (Mental health patients)	Corrigan et al. (1999) [36]	CTT			+			0	0		
Health Promotion Intervention Questionnaire (Mental health patients)	Svedberg et al. (2007) [33]	CTT							+		
Health Promotion Intervention Questionnaire (Mental health patients)	Svedberg et al. (2008) [32]	CTT		+	0		+	+	+		
Empowerment Questionnaire for Inpatients (Mental health patients)	Lopez et al. (2010) [37]	CTT		0	0		0	0	0		
Consumer Evaluation Of Mental Health Services (Mental health patients)	Oades et al. (2011) [34]	CTT		0			+++	0			
Inpatient Consumer Survey (Mental health patients)	Ortiz & Schacht (2012) [35]	CTT		0			0	+	0		
Diabetes Empowerment Scale version 1 (Diabetes patients)	Anderson et al. (2000) [16]	CTT		+			0	+	0		
Diabetes Empowerment Scale version 1 (Diabetes patients)	Sigurdardottir & Jonsdottir (2008) [41]	CTT		0			++	0	+	0	
Diabetes Empowerment Scale version 1 (Diabetes patients)	Tol et al. (2012) [42]	CTT		+			++	+	+	+	
Diabetes Empowerment Scale version 2 (Diabetes patients)	Shiu et al. (2003) [39]	CTT		+	0		++	+	+	+	
Diabetes Empowerment Scale version 2 (Diabetes patients)	Shiu et al. (2006) [40]	CTT		+				+	+		
Diabetes Empowerment Scale version 3 (Diabetes patients)	Leksell et al. (2007) [38]	CTT		+			0	+	0	0	
Chinese Diabetes Empowerment Process Scale (Diabetes patients)	Chen et al. (2011) [43]	CTT		+	+		+	+	0		
Patient Empowerment Scale (Cancer patients)	Bulsara et al. (2006) [17]	IRT	+	0			+	+			
Cyber Info-Decisional Empowerment Scale (Cancer patients)	Seckin (2011) [53]	CTT		+				+	+		
Genetic Counseling Outcome Scale (Clinical Genetics patients)	McAllister et al. (2011) [18]	CTT		0	+		++	++	0		+
Parents’ Postnatal Sense of Security (Postnatal patients)	Persson et al. (2007) [48]	CTT		+			+	+			
Psoriasis Empowerment Enquiry in the Routine practice (Psoriasis patients)	Pagliarello et al. (2010) [50]	CTT		+			0	+			
The Swedish Rheumatic Disease Empowerment Scale (Rheumatology patients)	Arvidsson et al. (2012) [51]	CTT		+			0	+	+		

COSMIN psychometric property boxes: A = internal consistency, B = reliability, C = measurement error, D = content validity, E = structural validity, F = hypothesis testing, G = cross-cultural validity, I = responsiveness. 4-point scale rating: +++ = excellent, ++ = good, + = fair, 0 = poor, empty space = COSMIN rating not applicable. IRT = item response theory; CTT = classical test theory.

For interpretability, only four studies reported how missing items were handled [18,34,35,44]. All four studies excluded responses with more than a minimum percentage of missing items. Only two of those studies reported methods of imputing responses where there were fewer than the minimum number of missing values [18,44]. Furthermore, only four studies reported the percentage of respondents with the highest possible score [38,47,49,51], and only three studies reported the percentage of respondents with the lowest possible score [38,41,51]. Similarly almost no studies reported on scores and change scores in sub-groups. Neither minimal important change (MIC) nor minimal important difference (MID) were assessed in any study.

For generalisability, most studies included patients with a wide age range. Distribution of sex seems to quite representative across all studies. Most studies were conducted in Western countries [13,16–18,28–30,32–38,41,44–53]. However, there were also several studies from Asia [31,39,40,42,43]. The most common sampling method was convenience sampling [13,16,37,38,43,47,52,53]; some studies did not report the sampling method at all [17,28,31,36,46,51] and only a few studies used randomised sampling [39,40,42].

### 5. Quality of instruments

The assessment of the quality of psychometric properties of included measures using the criteria developed by Terwee et al [23] is summarised in Table 5. It shows that content validity was assessed in 21 studies and received mainly positive or intermediate ratings. The Empowerment Scale was tested in five studies with only one of them testing content validity, assessed to be intermediate [36]. Internal consistency was tested in all but three studies [31,33,36], with mainly intermediate and positive ratings. Similarly, 24 studies reported on construct validity, with only a few negative ratings [35,37,38,51]. No information was found in any study on agreement. Only six studies assessed reliability, resulting in either intermediate [32,37] or positive [18,36,39,43] scores. Responsiveness, which was assessed in one study, received an intermediate score [18]. Information on floor and ceiling effects was reported in nine studies, resulting mainly in intermediate scores [28,37,41,46,47,52]. About half of the studies reported results that allowed rating of interpretability, and earned mainly intermediate scores [28,29,31,37,38,40,41,46,47,50–53]. The best performing measure identified in this study was the Chinese Diabetes Empowerment Process Scale, receiving positive scores for the four psychometric properties assessed. Content validity, internal consistency and construct validity were the most commonly investigated psychometric properties and results indicated intermediate to positive ratings. The psychometric property reproducibility (agreement) was never assessed.

**Table 5:** Quality of psychometric properties (Terwee ratings).

Measure (target population)	Authors (Year)	Content validity	Internal consistency	Construct validity	Agreement	Reliability	Responsiveness	Floor & ceiling effects	Interpretability
Patient Empowerment Scale (All patients, generic)	Faulkner (2001) [13]	+	-	0	0	0	0	0	0
Kim Alliance Scale (All patients, generic)	Kim et al. (2001) [46]	+	?	+	0	0	0	?	?
Kim Alliance Scale (Revised) (All patients, generic)	Kim et al. (2008) [47]	+	+	+	0	0	0	?	?
Treatment-Related Empowerment Scale (All patients, generic)	Webb et al. (2001) [45]	?	?	+	0	0	0	0	0
Health Education Impact Questionnaire (All patients, generic)	Osborne et al. (2007) [44]	+	+	0	0	0	0	0	0
No Name (Scale developed by Bann et al) (All patients, generic)	Bann et al. (2010) [49]	+	+	?	0	0	0	-	0
Health Care Empowerment Inventory (All patients, generic)	Johnson et al. (2012) [52]	0	?	+	0	0	0	?	?
Empowerment Scale version 1 (Mental health patients)	Wowra & McCarter (1999) [29]	0	+	?	0	0	0	0	?
Empowerment Scale version 1 (Mental health patients)	Hansson & Bjorkman (2005) [28]	?	-	+	0	0	0	?	?
Empowerment Scale version 1 (Mental health patients)	Yamada & Kuzuki (2007) [31]	0	0	?	0	0	0	0	?
Empowerment Scale version 1 (Mental health patients)	Rogers et al. (2010) [30]	0	-	?	0	0	0	0	0
Empowerment Scale version 2 (Mental health patients)	Corrigan et al. (1999) [36]	0	0	?	0	+	0	0	0
Health Promotion Intervention Questionnaire (Mental health patients)	Svedberg et al. (2007) [33]	0	0	+	0	0	0	0	0
Health Promotion Intervention Questionnaire (Mental health patients)	Svedberg et al. (2008) [32]	?	+	?	0	?	0	0	+
Empowerment Questionnaire for Inpatients (Mental health patients)	Lopez et al. (2010) [37]	+	?	-	0	?	0	?	?
Consumer Evaluation Of Mental Health Services (Mental health patients)	Oades et al. (2011) [34]	+	?	0	0	0	0	0	0
Inpatient Consumer Survey (Mental health patients)	Ortiz & Schacht (2012) [35]	+	+	-	0	0	0	0	0
Diabetes Empowerment Scale version 1 (Diabetes patients)	Anderson et al. (2000) [16]	-	+	?	0	0	0	0	0
Diabetes Empowerment Scale version 1 (Diabetes patients)	Sigurdardottir & Jonsdottir (2008) [41]	+	+	+	0	0	0	?	?
Diabetes Empowerment Scale version 1 (Diabetes patients)	Tol et al. (2012) [42]	?	?	?	0	0	0	0	0
Diabetes Empowerment Scale version 2 (Diabetes patients)	Shiu et al. (2003) [39]	+	+	?	0	+	0	0	0
Diabetes Empowerment Scale version 2 (Diabetes patients)	Shiu et al. (2006) [40]	0	+	?	0	0	0	0	?
Diabetes Empowerment Scale version 3 (Diabetes patients)	Leksell et al. (2007) [38]	0	-	-	0	0	0	+	?
Chinese Diabetes Empowerment Process Scale (Diabetes patients)	Chen et al. (2011) [43]	+	+	+	0	+	0	0	0
Patient Empowerment Scale (Cancer patients)	Bulsara et al. (2006) [17]	+	?	0	0	0	0	0	0
Cyber Info-Decisional Questionnaire (Cancer patients)	Seckin (2011) [53]	0	+	?	0	0	0	0	?
Genetic Counseling Outcome Scale (Clinical Genetics patients)	McAllister et al. (2011) [18]	+	?	?	0	+	?	0	0
Parents‘ Postnatal Sense of Security (Postnatal Care patients)	Persson et al. (2007) [48]	?	?	0	0	0	0	0	0
Psoriasis Empowerment Enquiry in the Routine practice (Psoriasis patients)	Pagliarello et al. (2010) [50]	+	-	0	0	0	0	0	?
The Swedish Rheumatic Disease Empowerment Scale (Rheumatology patients)	Arvidsson et al. (2012) [51]	+	-	-	0	0	0	-	?

Rating: + = positive, ? = intermediate, - = negative, 0 = no information available.

## Discussion

### 1. Contribution

Many PROMs have been developed to capture patient empowerment but when examined closely there is a diversity of definitions of patient empowerment captured by these measures. No two PROMs capture the same construct(s) of patient empowerment and existing tools have limited overlap in constructs captured. As a result, measurement of patient empowerment suffers from a lack of clarity and consensus about core constructs. Furthermore, the scientific quality of most instruments is low. Thirty studies were identified assessing 19 measures of patient empowerment that could be used as PROMs, the earliest of which was published in 1999.

In comparison to the Herbert et al review [19], the present review identified 21 new studies, with 15 new measures of patient empowerment, and added robust quality appraisal of included studies and measures using published quality criteria. Furthermore, the present study has classified the constructs captured by patient empowerment measures into four domains, supporting previous work suggesting that patient empowerment can be conceived of as a concept that is related to, but broader than patient-centeredness [54]. Patient empowerment can be conceptualised as a process achieved through patient-centered care, or as an outcome, and includes elements relating to both patient and healthcare professional roles, shared decision-making, patient self-efficacy and coping [54,55]. Constructs identified in domain 1 (patient states, experiences and capacities e.g. perceived control) and domain 2 (patient actions and behaviours e.g. health-directed behaviour) could be conceived of as patient outcomes from use of patient-centred healthcare. Constructs identified in domain 3 (patient self-determination within the healthcare relationship e.g. power-sharing and collaboration) and domain 4 (patient skills development) could be conceived of as the process through which patient and provider collaborate to achieve those outcomes [56].

Measures identified by Herbert et al. captured constructs ranging from psychological empowerment (interpersonal, interactional, behavioral), through knowledge, skills and attitudes, to decision-making, sense of control, hope, coping and self-efficacy. All of these constructs could be identified in measures included in the present study, with additional dimensions captured by some of the newer measures e.g. acceptance [17], emotional wellbeing/regulation [18,44], tolerance of uncertainty [52], stigma [34] and sense of affinity with the family [48]. There is also some cultural diversity identifiable with stress management included only in Swedish measures, and some constructs that may be specific to the mental health context. This variation appears to reflect lack of clear conceptualisation of patient empowerment. While there is some overlap across these constructs, the heterogeneity of terms used to describe patient empowerment appears to be increasing with time and is not helpful for measurement. This also limits the comparability of findings across surveys as different measures capture different aspects of patient empowerment, based upon their different conceptual underpinnings. The variation in constructs captured by patient empowerment questionnaires has been noted previously [12,19], yet members of this team were surprised at the extent of variation when limiting this review to focus only on instruments that aim to measure patient empowerment. Without conceptual clarity of what patient empowerment is, measurement tools will inevitably vary and choosing tools to assess patient empowerment either as a process or outcome will be difficult.

There is considerable overlap between the constructs captured in the measures identified in this review and constructs captured by other measures not purporting to be measures of patient empowerment, such as enablement, activation, shared decision-making and capability. The Patient Enablement Instrument focuses on whether patients feel able to understand their illness, cope with their illness and their lives, and keep themselves healthy [57]. The Patient Activation Measure (PAM) [58] is based on a developmental model, capturing patient development across four stages: (1) believing the patient role is important, (2) having the confidence and knowledge necessary to take action, (3) actually taking action to maintain and improve one’s health, and (4) staying the course even under stress [58]. Both enablement and activation overlap with some, but not all constructs across the four domains identified in the current study. However, they may not adequately capture all dimensions of patient empowerment.

Other areas of cross over with patient empowerment include measures of shared decision-making, which may capture aspects of collaboration and mutual decision-making, components of domain 3 in our categorisation of patient empowerment [59,60]. The ICECAP capability [61,62] measures capture patient wellbeing defined in terms of ability to 'do' and 'be' the important things in life: enjoyment, achievement and attachment and is a more general measure of patient quality of life. There are also a number of measures available to capture self-efficacy, perceived control and other constructs captured by measures identified in the current study [63,64]. Some or all of these may be better quality measures in respect of their psychometric properties, but a full review of these is outside the scope of the present review which focused on measures purporting to capture patient empowerment.

The quality of included studies was found to be poor to fair, with many psychometric properties of instruments untested. Where these properties were tested, there was limited evidence to support reliability and validity of existing measures. Some elements of COSMIN may not be applicable to all studies, for example only five of 30 studies were assessed for cross-cultural validity, as they had translated a measure from the original language. However, it is of some considerable concern that few studies investigated measurement error, reliability, criterion validity and responsiveness. Of particular concern is the lack of reliability assessment, as there can be no validity of measurement without reliability. When assessing reliability it is recommended that both internal consistency and test-retest reliability be investigated [65]. We found that many authors used internal consistency as the sole indication of reliability; this is an inadequate assessment of reliability [65].

From a policy and practice standpoint, for a measure to be useful as a PROM for evaluating interventions, it is vital to understand how responsive the measure is to change in the underlying construct being measured e.g. use of a decision support tool to promote patient empowerment. However, only one measure identified in this review was assessed for responsiveness, the Genetic Counseling Outcome Scale [18], which received an intermediate rating. It is also pertinent to have some indication of the minimal important change (MIC) or minimal important difference (MID) in scores on the measure (COSMIN v9). Without such insight, it is not possible to understand whether changes in levels of patient empowerment matter to patients. No study included in the present review assessed the MID or MIC of the measure under investigation.

The World Health Organisation [1] called for better measures of patient empowerment in 2006. However, although the three most commonly assessed psychometric properties of the measures identified in this review (content validity, internal consistency and construct validity) earned intermediate to positive ratings, other important properties, notably reliability and responsiveness were not assessed for most measures. No study assessed all the psychometric properties highlighted by Terwee et al [23]. Without full assessment of psychometric properties, the validity and reliability of results generated by use of that measure are questionable. Another significant concern was the variation in quality of the psychometric properties of the same measure when assessed across different studies. For example internal consistency of the Empowerment Scale earned a rating of ‘positive’ in one study [29], ‘intermediate’ in second study [31] and ‘poor’ in two other studies [28,30].

The findings of limited psychometric quality of the 19 questionnaires identified in this study designed to capture patient empowerment is similar to that found in the Herbert systematic review [19] of measures of health-related empowerment. Interestingly test-retest reliability was assessed in five of the 50 studies identified by Herbert et al [19] and only one of 50 studies reported good evidence of validity and reliability for the measures under investigation. Of those, only four had moderate support. The challenges facing the field of patient empowerment are similar to those in related fields, such as shared decision-making [59,60] and decisional regret [66], where lack of conceptual clarity and limited evidence of reliability and validity of measures are well-documented. These constructs are similar in that they are not directly observable (latent), multidimensional and most importantly, are related to patients and their important, but subjective perceptions.

### 2. Strengths and limitations

This is the first systematic review of measures of patient empowerment to apply published quality criteria, assessing both the methodological quality of the studies and the psychometric properties of the measures identified. This review enables researchers and clinicians to view at a glance the strengths and limitations of existing measures of patient empowerment that could be used as PROMs in terms of constructs captured and psychometric properties. Despite the strengths of the review, there are some limitations. Firstly the method deviated from the protocol when a decision was made to exclude measures of enablement, activation, perceived control, capability and independence and to focus only on measures that aim to capture patient empowerment. However, a review of the 102 articles identified that included those reporting measures capturing these other five constructs would not have included all measures that capture similar / overlapping constructs (e.g. all measures of self-efficacy). Limiting the review to measures purporting to capture patient empowerment enabled a clear focus on patient empowerment to develop understanding of how this construct has been operationalised in measures purporting to capture it as well as establishing the quality of these measures, while removing the ambiguity of including measures of related, but subtly different constructs. As with any systematic review, there is a lag between the time of the search completion and the final manuscript publication; therefore it is possible that we may have missed more recent literature. For example, a recently developed new measure of empowerment for use in long-term conditions was developed by Small et al [14]. This measure captured a construct of empowerment comprising positive attitude and sense of control, knowledge and confidence in decision making and enabling others. Psychometric testing was limited to assessment of structural validity, internal consistency and hypothesis testing, with no assessment of either test-retest reliability or responsiveness.

### 3. Implications for future research

This systematic review adds to the findings of Herbert et al [19] and highlights the need for a definitive measure of patient empowerment. Despite policy interest and initiatives relating to patient empowerment, there is limited evidence to support the reliability and validity of existing tools. Future research could usefully develop a definitive generic measure of patient empowerment—one that is valid, reliable and sensitive to changes important to patients and other stakeholders, for use across healthcare systems to enable comparability of results.

This systematic review belongs mainly in the positivist tradition, incorporating a partly constructivist approach, recognising multiple interpretations, in the analysis of constructs captured by the included measures. We believe that moving forward, a combination of constructivist and critical realist positions will be most productive in patient empowerment research. This approach would be in keeping both with (1) ensuring development of a construct to operationalise in the new measure that has consensual support amongst key stakeholders and with (2) the emerging ‘realist’ approach to evaluating complex social interventions or programmes, which focuses on establishing what works for whom and how this can best be achieved [67]. This approach reflects a belief that patient empowerment does have an underlying structure that could be partly revealed through (constructivist) Qualitative Item Review (QIR) [68] of items captured in measures of patient empowerment identified in the current review, supplemented by (constructivist) qualitative research with patients and a (constructivist/realist) consensus exercise amongst stakeholders, soliciting views from patients, health providers and healthcare policy makers. This epistemological shift would make it possible to develop a valid measure of patient empowerment that captures a construct that is plausible and important to a range of stakeholders. The constructs identified in the present review as captured by at least two measures provide a useful starting point for this work.

### 4. Conclusions

This study has identified significant effort over the last 25 years to develop innovative measures that aim to capture patient empowerment. This reflects a surge of interest in the idea of empowering patients, and of measuring the degree to which this can be demonstrated. However, the review has highlighted significant shortcomings of available measures of patient empowerment, particularly for use as PROMs in evaluating healthcare policies and interventions. Available measures capture a diversity of constructs and have very limited evidence of two psychometric properties that are vital for PROMs, reliability and responsiveness, and no available measures have been tested for MID or MIC, also very important for any measure that is to be used as a PROM. This study contributes significant clarification of how patient empowerment, as operationalised in measures purporting to capture this concept, overlaps with, and differs from other related constructs. More research is needed to develop a clear definition of patient empowerment that could be operationalised to create a definitive, valid, reliable and responsive measure of patient empowerment. A definitive PROM capturing patient empowerment would enable healthcare interventions and policies designed to empower patients to be evaluated on the basis of how effective they are at achieving that goal.

## Supporting Information

S1 Fig Electronic search strategies: Empowerment searches. (PDF)

S1 PRISMA Checklist PRISMA checklist. (PDF)

S1 Table Detailed results for the COSMIN checklist with 4-point scale rating. (PDF)
