# Supplementary material for: Assessment of Patient Empowerment - A Systematic Review of Measures
Source: PLoS One. 2015 May 13;10(5):e0126553. doi: 10.1371/journal.pone.0126553 (PMC4430483; doi:10.1371/journal.pone.0126553)
Supplement: S1 Fig — (PDF) [file pone.0126553.s001.pdf]

Supplementary information S1 Figure: Electronic search strategies: Empowerment searches

Ovid- Medline

1. exp psychometrics/
2. exp factor analysis, statistical/
3. factor structure.mp.
4. item reduction.mp.
5. valid$.mp.
6. internal consistency.mp.
7. test-retest.mp.
8. kappa.mp.
9. intraclass correlation.mp.
10. ceiling effect$.mp.
11. floor effect$.mp.
12. item response model.mp.
13. item response theory.mp.
14. rasch analysis.mp.
15. differential item function.mp.
16. cross-cultural validation.mp.
17. exp "Reproducibility of Results"/ or exp Observer Variation/ or inter rater reliability.mp.
18. reliability.mp.
19. ("intra rater reliability" or "intrarater reliability").mp.
20. measurement error$.mp.
21. Standard Error of Measurement.mp.
22. Smallest Detectable Change.mp.
23. Limits of Agreement.mp.
24. face validity.mp.
25. factor analysis.mp.
26. convergent validity.mp.
27. criterion validity.mp.
28. receiver operating curve.mp.
29. exp ROC Curve/ or ROC.mp.
30. responsiveness.mp.
31. exp "Sensitivity and Specificity"/
32. Cronbach$.mp.
33. or/1-32
34. exp Health Surveys/ or "health survey$".tw.
35. exp Questionnaires/ or questionnair$.tw.
36. exp interview/ or interview$.tw.

37. survey$.tw.
38. exp Psychological Tests/ or "psychological test$".tw.
39. measure$.ab,ti.
40. scale$.ab,ti.
41. self report.ab,ti.
42. self reported.ab,ti.
43. test.ab,ti.
44. tests.ab,ti.
45. testing.ab,ti.
46. exp "Outcome Assessment (Health Care)"/
47. or/34-46
48. exp "Power (Psychology)"/ or power.ti.
49. empower$.ab,ti.
50. enablement.ab,ti.
51. activation.ab,ti.
52. personal control.ab,ti.
53. or/48-52
54. health.ab,ti.
55. exp Health/
56. exp Family/
57. family.ab,ti.
58. consultation.ab,ti.
59. exp Patients/
60. exp "Patient Acceptance of Health Care"/
61. exp Patient Education as Topic/
62. exp Patient Satisfaction/
63. exp Professional-Patient Relations/
64. patient.ab,ti.
65. or/54-64
66. 33 and 47 and 53 and 65

WOS

TDC_Empowerment_Barr Show Details

Results Topic=(empower* OR autonomy OR "personal control" OR enablement) AND Topic=(test OR test OR questionnaire* OR interview* OR instrument* OR measurement*) AND Topic=(PATIENT* OR MEDICAL OR MEDICAL) AND Topic=(VALID* OR FACTOR* OR ANALYS* OR ITEM OR AGREEMENT OR RASCH OR KAPPA OR RETEST OR CRITERION OR REPRODUC*)
Refined by: [excluding] Databases=(MEDLINE)
Timespan=All Years.
Search language=Auto Lemmatization=On

	empower* OR autonomy OR "personal control" OR enablement<br>*Example:* oil spill* mediterranean	in	Topic
AND	test OR test OR questionnaire* OR interview* OR instrument* OR measurement*<br>*Example:* oil spill* mediterranean	in	Topic
AND	PATIENT* OR MEDICAL OR MEDICAL<br>*Example:* oil spill* mediterranean	in	Topic
AND	VALID* OR FACTOR* OR ANALYS* OR ITEM OR AGREEMENT OR RASCH OR KA<br>*Example:* oil spill* mediterranean	in	Topic

ASSIA

TDC_Empowerment_Barr Show Details

(SU.EXACT.EXPLODE("Disempowerment" OR "Empowerment" OR "Psychological empowerment" OR "Selfempowerment") OR all(empower* OR autonomy OR "personal control")) AND all((test OR tests OR questionnaire* OR interview* OR instrument* or measurement*)) AND all(patient* OR medical OR medicine) AND all(valid* OR analyst* OR agreement OR rasch OR kappa OR retest OR criterion R reproduce*)

EMBASE

#13	#12 AND [embase]/lim	935
#12	#3 AND #4 AND #9 AND #11	1,111
#11	'health'/exp OR 'family'/exp OR 'patient'/exp OR 'patient attitude'/exp OR 'patient satisfaction'/exp OR 'patient education'/exp	1,644,951
#9	#7 OR #8	1,126,230*
#8	power OR empower* OR enablement OR activation OR 'personal control'	1,125,835
#7	'empowerment'/exp	2,053
#4	'health survey'/exp OR 'questionnaire'/exp OR 'interview'/exp OR 'psychologic test'/exp OR 'outcome assessment'/exp	872,601
#3	#1 OR #2	1,106,276
#2	'psychometrics'/exp OR 'factor analysis'/exp OR 'factor structure' OR 'item reduction' OR valid* OR 'internal consistency'/exp OR 'test-retest' OR kappa OR 'intraclass correlation' OR 'ceiling effect' OR 'floor effect' OR 'item response' OR rasch OR 'differential item' OR 'cross-cultural valid' OR 'reproducibility'/exp OR 'reliability'/exp OR intrarater OR interrater OR 'measurement error'/exp OR 'standard error' OR 'smallest detectable' OR 'limits of agreement' OR 'face validity'/exp OR 'convergent validity' OR 'criterion validity' OR 'receiver operating curve' OR roc OR responsiveness OR cronbach*	842,315
#1	'psychometry'/exp OR 'factorial analysis'/exp OR 'validity'/exp OR 'internal consistency'/exp OR 'correlation analysis'/exp OR 'reproducibility'/exp OR 'receiver operating characteristic'/exp OR 'sensitivity and specificity'/exp OR 'test retest reliability'/exp OR 'interrater reliability'/exp OR 'intrarater reliability'/exp	537,679

Cochrane

TDC_Empowerment_Barr Show Details

Search Name:
Date Run: 02/11/12 19:37:21.617
Description:

ID Search Hits
#1 test or test or questionnaire* or interview* or instrument* or measurement* 216491
#2 empower* or autonomy or "personal control" or enablement or empowerment 1162
#3 #1 and #2 775
#4 empower* or autonomy or "personal control" or enablement or empowerment:ti (Word variations have been searched) 182
#5 #1 and #4 86
#6 MeSH descriptor: [Power (Psychology)] explode all trees 126
#7 #1 and #6 72
#8 #5 or #7 130
