# Supplementary material for: Assessment of Patient Empowerment - A Systematic Review of Measures
Source: PLoS One. 2015 May 13;10(5):e0126553. doi: 10.1371/journal.pone.0126553 (PMC4430483; doi:10.1371/journal.pone.0126553)
Supplement: S1 Table — (PDF) [file pone.0126553.s003.pdf]

Supplementary Information S2 Table: Detailed results for the COSMIN checklist with 4-point scale rating

*COSMIN psychometric properties/Items**	*study IDs/Rating scores*																											
**IRT**																	**S19**									**S28**		
Was the IRT model used adequately described?																	+++									+++		
Was the software package used adequately described?																	+++									+++		
Was the method of estimation used adequately described?																	+++									++		
Were the assumptions for estimating parameters of the IRT model checked?																	++									+		
**Internal consistency**	**S1**	**S2**	**S3**	**S4**	**S5**	**S6**	**S7**	**S9**	**S10**	**S11**	**S12**	**S13**	**S14**	**S16**	**S17**	**S18**	**S19**	**S20**	**S21**	**S22**	**S23**	**S24**	**S25**	**S26**	**S27**	**S28**	**S29**	
1. Does the scale consist of effect indicators, i.e. is it based on a reflective model?*	ok	ok	ok	ok	ok	ok	ok	ok	ok	ok	ok	ok	ok	ok	ok	ok	ok	ok	ok	ok	ok	ok	ok	ok	ok	ok	ok	
2. Was the percentage of missing items given?	++	++	++	++	++	++	++	++	++	++	++	++	++	++	++	++	+++	++	0	++	++	++	++	++	++	++	++	
3. Was there a description of how missing items were handled?	+	+	+	+	+	+	+	+	+	+	+	+	+	+	+	+++	+++	+	+++	+++	+	+	+	+	+	+	+	
4. Was the sample size included in the internal consistency analysis adequate?	+++	++	+++	++	+++	+++	++	+++	+++	+++	+++	++	+++	+++	+++	+++	+++	+++	+++	+++	+++	+	+++	+++	+++	+++	+++	
5. Was the unidimensionality of the scale checked? I	0	+++	+++	+++	+++	+++	+++	+++	+++	+++	+++	0	+++	+++	+++	+++	+++	+++	+++	+++	+++	0	+++	+++	+++	+++	+++	
6. Was the sample size included in the unidimensionality analysis adequate?	n/a	0	+++	++	+++	+++	+	++	+++	+++	+++	0	+++	+++	+++	0	+++	+++	+++	++	+	0	+++	+++	+++	0	+++	
7. Was an internal consistency statistic calculated for each (unidimensional) (sub)scale separately?	+++	+++	+++	+++	+++	+++	0	+++	+++	+++	+++	+++	+++	+++	+++	+++	+++	+++	+++	0	+++	0	+++	+++	+++	n/a	+++	
8. Were any important flaws in the design or methods of study?	+++	+++	++	+++	+++	+++	0	+	+++	+++	+++	+++	+++	+++	0	+++	0	+++	+	+++	+	0	+++	+++	+++	+++	+++	
9. For Classical Test Theory (CTT): Continuous scale: Was Cronbach's alpha included?	+++	+++	++	+++	+++	+++	+++	+++	+++	+++	+++	+++	+++	+++	+++	+++	+++	+++	+++	+++	+++	+++	+++	+++	+++	n/a	+++	
10. For CTT: Dichotomous scores: Was Cronbach's alpha or KR-20 calculated?	n/a	n/a	n/a	n/a	n/a	n/a	n/a	n/a	n/a	n/a	n/a	n/a	n/a	n/a	n/a	n/a	n/a	n/a	n/a	n/a	n/a	n/a	n/a	n/a	n/a	n/a	n/a	
11. For IRT: was a goodness of fit statistic at a global level calculated?	n/a	n/a	n/a	n/a	n/a	n/a	n/a	n/a	n/a	n/a	n/a	n/a	n/a	n/a	n/a	n/a	n/a	n/a	n/a	n/a	n/a	n/a	n/a	n/a	n/a	+++	n/a	
**Final score Box A**	0	0	+	+	+	+	0	+	+	+	+	0	+	+	0	0	0	+	0	0	+	0	+	+	+	0	+	
**Reliability**							**S7**				**S12**		**S14**							**S22**							**S29**	**S3[illegible]**
1. Was the percentage of missing items given missing?							++				++		++							++							++	++
2. 'Was there a description of how missing items were handled?							+				+		+							+++							+	+

3. Was the sample size included in the analysis adequate?							0					0		0								+++							+	+
4. Were at least two measurements available?							+++					+++		+++								+++							+++	+++
5. Were the administrations independent?							+					+++		+++								+++							+	+
6. Was the time interval stated?							+++					+++		+++								+++							+++	+++
7. Were the patients stable in the interim period on the construct to be measured?							++					++		++								++							+	+
8. Was the time interval appropriate?							++					+++		0								+							+++	+++
9. Were the test conditions similar for both measurements?							+					++		++								++							+	+
10. Were any important flaws in the design or methods of study?							0					+++		+++								+							+++	+++
11. For continuous scores: Was an intraclass correlation coeffecient (ICC) calculated							+					+++		n/a								+++							++	+
12. For dichotomous/nominal/ordinal scores: Was Kappa calculated?							0					n/a		+++								n/a							n/a	n/a
13. For ordinal scores: Was the weighting scheme distributed?							n/a					n/a		+++								n/a							n/a	n/a
**Final score Box B**							0					0		0								+							+	+
**Measurement error**																														
**Content validity**	**S1**	**S2**	**S3**	**S4**	**S5**	**S6**	**S7**		**S9**			**S12**	**S13**	**S14**				**S18**	**S19**	**S20**	**S21**	**S22**	**S23**	**S24**	**S25**	**S26**	**S27**	**S28**	**S29**	
1. Was there an assessment of whether all items refer to relevant aspects of construct to be measured?	+++	+++	+++	+++	+++	0	+++		+++			+++	+++	+++				+++	+++	+++	+++	+++	+++	+	+	+++	+++	+++	+++	
2. Was there an assessment of whether all items are relevant for the study population?	+++	0	0	0	0	0	++		+++			+++	+++	+++				+++	+++	0	0	++	+	+	0	+++	+++	+++	+++	
3. Was there an assessment of whether all items are relevant for the purpose of the measurement instrument?	+	+	+	+++	+++	0	+		++			++	++	++				+++	+++	+++	+++	+++	++	+	+	+	+	+	+	
4. Was there an assessment of whether all items together comprehensively reflect the construct to be measured?	0	+++	+	+++	+++	0	+		+++			+++	+++	+				+++	+++	+	+	+++	+++	0	0	0	0	+++	+++	
5. Were any important flaws in the design or methods of study?	+++	0	0	+++	+++	+++	0		+++			+++	+++	+++				+++	+	+++	+++	+++	+++	0	+++	+++	+++	+++	+++	
**Final score Box D**	0	0	0	0	0	0	0		++			++	++	+				+++	+	0	0	++	+	0	0	0	0	+	+	
**Structural validity**		**S2**	**S3**	**S4**	**S5**	**S6**	**S7**	**S8**	**S9**	**S10**	**S11**	**S12**	**S13**	**S14**		**S16**	**S17**	**S18**	**S19**	**S20**	**S21**	**S22**	**S23**		**S25**	**S26**	**S27**	**S28**	**S29**	**S3**
1. Does the scale consist of effect indicators, i.e. is it based on a reflective model?		ok	ok	ok	ok	ok	ok	ok	ok	ok	ok	ok	ok	ok		ok	ok	ok	ok	ok	ok	ok	ok		ok	ok	ok	ok	ok	ok
2. Was the percentage of missing items given?		++	++	++	++	++	++	++	++	++	++	++	++	++		++	++	++	+++	++	+++	+++	++		++	++	++	++	++	++
3. Was there a description of how missing items were handled?		+	+	+	+	+	+	+	+	+	+	+	+	+		+	+	+++	+++	+	+++	+++	+		+	+	+	+	+	+
4. Was the sample size		0	+++	++	+++	+++	+	0	++	+++	+++	+++	0	+++		+++	+++	0	+++	+++	+++	++	++		+++	+++	+++	+++	+++	0

included in the analysis adequate?																													
5. Were any important flaws in the design or methods of study?	+	+++	+	+	+	+	+	+++	+++	+++	+++	+++	+++		+++	0	+	+	+	+	+++	+		+++	+++	+++	+++	+++	+++
6. For CTT: Was exploratory or confirmatory factor analysis performed?	+++	+++	+++	+++	+++	0	0	+++	+++	+++	+++	0	+++		+++	+++	+++	n/a	+++	+++	+++	+++		+++	++	++	n/a	+++	+++
7. For IRT: Were IRT tests for determining the (uni-) dimensionality of items performed?	n/a	n/a	n/a	n/a	n/a	n/a	n/a	n/a	n/a	n/a	n/a	n/a	n/a		n/a	n/a	n/a	+++	n/a	n/a	n/a	n/a		n/a	n/a	n/a	+++	n/a	n/a
**Final score Box E**	0	+	+	+	+	0	0	+	+	+	+	0	+		+	0	0	+	+	+	++	+		+	+	+	+	+	0

**Hypotheses testing**	**S2**	**S3**	**S4**	**S5**	**S6**	**S7**	**S8**	**S9**	**S10**	**S11**	**S12**	**S13**	**S14**	**S15**	**S16**	**S17**				**S21**	**S22**		**S24**	**S25**	**S26**	**S27**		**S29**	**S30**
1. Was the percentage of items given missing?	++	++	++	++	++	++	++	++	++	++	++	++	++	++	++	++				+++	++		++	++	++	++		++	++
2. Was there a description of how missing items were handled?	+	+	+	+	+	+	+	+	+	+	+	+	+	+	+	+				+++	+++		++	+	+	+		+	+
3. Was the sample size included in the analysis adequate?	0	+	0	0	0	0	0	+++	+++	+++	+++	+	+	+	++	+++				+++	+++		+	+	++	+++		0	0
4. Were hypotheses regarding correlations or mean differences formulated a priori	+++	+	+++	++	+++	+	0	+	+	+	++	+++	+	+++	+	0				+	+		+++	0	++	+		++	+++
5. Was the expected direction of correlations or mean differences included in the hypotheses?	+++	++	+++	++	+++	++	++	++	++	++	+++	+++	++	+++	++	++				++	++		+++	++	+++	++		++	+++
6. Was the expected absolute or relative magnitude of correlations or mean differences include in the hypotheses?	++	++	++	++	++	++	++	++	++	++	++	++	++	++	++	++				++	++		++	++	++	++		++	++
7. For convergent validity: Was an adequate description provided of the comparator instrument(s)?	+++	++	+	0	+++	++	+++	n/a	n/a	n/a	n/a	+++	n/a	+++	n/a	+++				0	+		+++	++	n/a	+++		+++	+++
8. For convergent validity: Were the measurement properties of the comparator instrument(s) adequately described	+	+	++	0	0	+	+	n/a	n/a	n/a	n/a	+++	n/a	+++	n/a	++				0	0		+++	+	n/a	+++		++	++
9. Were any important flaws in the design or methods of study?	+	+	+	+	+	+	+	+++	+	+++	+	+	+++	+++	+++	0				0	+++		0	+	+	+		+	+++
10. Were design and statistical methods adequate for the hypotheses to be tested?	+++	+++	+++	+++	+++	+++	+++	+++	+	+++	+++	+++	+++	+++	+++	++				++	++		+++	+	+++	+++		+++	++
**Final score Box F**	0	+	0	0	0	0	0	+	+	+	+	+	+	+	+	0				0	0		0	0	+	+		0	0

**Cross-cultural validity**	**S2**	**S6**	**S9**	**S12**	**S13**
1. Was the percentage of items given missing?					
2. Was there a description of how missing items were handled?	++	++	++	++	++
3. Was the sample size included in the analysis adequate?	+	+	+	+	+
4. Were both the original language in which the HR-PRO instrument was	0	+++	++	+++	0

developed, and the language in which the HR-Pro instrument was translated described? (which language?)								
5. Was the expertise of the people involved in the translation process adequately described?	0	+++	+++	+++	+++			
6. Did the translators work independently from each other?	+	++	+++	+++	+++			
7. Were the items translated forward and backward?	+	+	+	+	+			
8. Was there an adequate description of how differences between the original and translated versions were resolved?	0	+	+	+	+			
9. Was the translation reviewed by a committee	++	++	++	++	++			
10. Was the HR-PRO instrument pre-tested (e.g. cognitive interviews) to check interpretation, cultural relevance of the translation, and ease of comprehension?	++	++	+++	+++	+++			
11. Was the sample used in the pre-test adequately described?	++	0	++	++	+++			
12. Were the samples of similar for all characteristics except language and/or cultural background?	+	n/a	+	+	+			
13. Were any important flaws in the design or methods of study?	+	+	+	+	+			
14. For CTT: Was confirmatory factor analysis performed? (Please record findings)	+++	+	+++	+++	+++			
15. For IRT: Was differential item function (DIF) between language groups assessed?	n/a	n/a	n/a	n/a	n/a			
**Final score Box G**	0	0	+	+	0			
**Criterion validity**							0	+
**Responsiveness**						**S22**		
1. Was the percentage of items given missing?						+++		
2. Was there a description of how missing items were handled?						+++		
3. 'Was the sample size included in the analysis adequate?						+++		
4. Was a longitudinal design with at least two measurement used?						+++		
5. Was the time interval stated?						+++		
6. If anything occurred in the interim period (e.g intervention, other relevant events) was it adequately						+		

described?	
7. Was a proportion of the patients changed (i.e improvement or deterioration)?	+
For constructs for which a gold standard was not available	
8. Were hypotheses about changes in scores formulated a priori (i.e before data collection)?	+
9. Was the expected direction of correlations or mean differences of the change scores of HR-PRO instruments included in these hypotheses?	++
10. Were the expected absolute or relative magnitude of correlations or mean differences of the change scores of HR-PRO instruments included in these hypotheses?	++
11. Was an adequate description provided of the comparator instrument(s)? (Please describe comparator)	n/a
12. Were the measurement properties of the comparator instrument(s) adequately described?	n/a
13. Were any important flaws in the design or methods of study?	+++
14. Were the design and statistical methods adequate for the hypotheses to be tested?	+
Final score Box I	+

* Description of item content altered to fit this table. For exact item content see COSMIN website (www.cosmin.nl). Study IDs: S1: Faulkner, 2001; S2: Hansson & Bjorkman, 2005, Sweden; S3: Johnson et al., 2012; S4 Kim et al., 2001; S5: Kim et al., 2008; S6: Leksell et al., 2007; S7: Lopez et al., 2010; S8: Yamada et al, 2007; S9: Tol et al, 2012; S10: Seckin et al, 2011; S11: Shiu et al, 2006; S12: Shiu et al, 2003; S13: Sigurdardottir et al, 2008; S14: Svedberg et al, 2008; S15: Svedberg et al, 2007; S16: Wowra et al, 1999; S17: Rogers et al, 2010; S18: Oades et al, 2011; S19: Osborne et al, 2007; S20: Pagliarello et al, 2010; S21: Ortiz & Schacht, 2012; S22: McAllister et al, 2011; S23: Persson et al, 2007; S24: Webb et al. 2001; S25: Anderson et al. 2000; S26: Arvidsson et al. 2012; S27: Bann et al. 2010; S28: Bulsara et al. 2006; S29: Chen e al. 2011; S30: Corrigan et al, 1999. 4-point scale rating: +++ = excellent, ++ = good, + = fair, 0 = poor, empty space = COSMIN rating not applicable. n/a = not applicable.
